# Ecological Stoichiometry and Microbial Community Dynamics in the Rhizosphere of *Parashorea chinensis*: Implications for Ex Situ Conservation of Endangered Trees

**DOI:** 10.3390/plants15213275

**Published:** 2026-10-23

**Authors:** Wannian Li, Yujun Liu, Ting Li, Yuanyuan Xu, Xianyu Yao, Mei Yang

**Affiliations:** 1 Guangxi Colleges and Universities Key Laboratory for Cultivation and Utilization of Subtropical Forest Plantation, School of Forestry, Guangxi University, Nanning 530004, China; liwannianok@163.com (W.L.); yuanyuanxu_2016@163.com (Y.X.); 2 Qinghai Haibei National Field Research Station of Alpine Grassland Ecosystem, Northwest Institute of Plateau Biology, Chinese Academy of Sciences, Xining 810008, China; 3 Nanning Arboretum, Nanning 530031, China; 13307714068@189.com (Y.L.); catlee0601@163.com (T.L.)

**Keywords:** *Parashorea chinensis*, ex situ conservation, growth stages, rhizosphere soil microorganisms, ecological stoichiometry

## Abstract

Ex situ conservation of the endangered tree *Parashorea chinensis*, a national first-class protected species in China, hinges on the development of functional belowground ecosystems, a process that remains mechanistically unclear in restoration ecology. This study evaluated the development of conservation plantations by analyzing rhizosphere soil stoichiometry, enzyme activities, and microbial communities across four growth stages in natural forests and ex situ plantations. Results showed that ex situ plantations exhibited obvious soil acidification and persistently low N:P ratios, with phosphorus acting as the primary limiting nutrient. Fungal α-diversity was strongly correlated with stoichiometric imbalance in ex situ plantations. Artificial management drove microbial community shifts toward fungal-enriched assemblages, forming a distinctive functional microbial portfolio to tolerate combined stresses from acidification and nutrient stoichiometric imbalance. Crucially, these managed ecosystems maintain core nutrient cycling functions, and key microbial indicators show distinct developmental trends toward the conditions observed in natural forests as stands mature. In summary, ex situ conservation alters belowground biogeochemical processes rather than merely supporting plant survival. We propose targeted microbial management, particularly inoculation with native functional taxa from natural forests, to accelerate soil stoichiometry rebalancing and enhance nutrient cycling efficiency. These findings provide both evidence for the ecological feasibility of ex situ conservation and practical strategies for optimizing its long-term success through science-based belowground management.

## 1. Introduction

The conservation of endangered tree species via ex situ measures constitutes an essential approach to preserving global biodiversity [1], yet its practical execution remains fraught with significant difficulties. A growing body of evidence suggests that many threatened tree species are predominantly associated with low-biomass plant communities [2], and their decline is commonly linked to elevated ecosystem productivity driven by nutrient enrichment. Although nitrogen (N) deposition has long been implicated as a primary agent of biodiversity loss [3], emerging insights underscore that phosphorus (P) enrichment may exert comparable or even more critical effects in certain contexts. Notably, species classified on the Red List have demonstrated greater persistence in P-limited habitats than in N-limited environments [4,5], with many having vanished from over 20% of their documented ranges within just two decades. This predominant focus on N, however, may be indicative of a broader geographic bias in ecological research, particularly toward temperate regions where nitrogen limitation is more commonly reported [6]. Consequently, a more nuanced understanding of N-P stoichiometry and its physiological implications is urgently needed. The synergistic or antagonistic interactions between nitrogen and phosphorus availability not only regulate plant fitness and competitive outcomes but may also determine the long-term viability of conservation efforts for endangered species, largely through mediating belowground biotic processes.

Nutrient stoichiometry, especially the nitrogen-to-phosphorus (N:P) ratio, serves as a fundamental constraint on plant community assembly and ecosystem functioning [7]. These stoichiometric constraints operate in part through microbial mediation; imbalances in soil N and P availability affect extracellular enzyme activities [8,9], microbial community composition, and ultimately the mineralization rates of key nutrients [10]. Such microbial feedback can either facilitate or hinder nutrient acquisition by plants, particularly under ex situ conservation conditions, where soil properties are often artificially managed. Importantly, many global biodiversity hotspots occur in tropical regions characterized by ancient, highly weathered soils where phosphorus is frequently the primary limiting nutrient [11,12,13]. Within these ecosystems, the mechanisms through which nutrient enrichment drives vegetation shift are likely distinct from those in temperate, N-limited systems. It follows that effective ex situ conservation strategies must account for such biogeographical differences in nutrient limitation [14], yet the role of soil microbiomes in mediating these responses remains critically understudied. Therefore, clarifying how soil N:P stoichiometry and associated microbial processes influence the performance of endangered trees in artificial settings is essential to improving conservation outcomes.

*Parashorea chinensis*, a critically endangered canopy tree endemic to tropical rainforests of China [15], offers an instructive model system to examine these mechanisms. Although ex situ plantings have been established, their success is often compromised by nutrient-acquisition challenges and arrested growth, pointing to a potential breakdown in the beneficial root–soil–microbe feedback essential for fitness in its native habitat [16]. What remains especially unclear is how these stoichiometric and microbial interactions shift across the tree’s developmental stages in planted stands. Nutrient limitations that initially suppress seedling establishment may differ from those affecting sapling or mature tree performance. A chronosequence study of artificial plantations across different ages is thus needed to unravel temporal dynamics in nutrient limitation and microbial community co-development. Such an approach would not only illuminate stage-specific nutrient demands but also provide actionable insights for tailoring nutrient and microbial management during ex situ population development.

Against this backdrop, most conservation studies of *P. chinensis* focus primarily on aboveground performance, ignoring coupled dynamics of soil stoichiometric imbalance and rhizosphere microbes across growth stages, and few compare rhizosphere soil–microbe relationships between natural primary forests and ex situ plantations. We compare rhizosphere stoichiometry, extracellular enzyme activities and microbial communities across developmental stages in natural and planted *P. chinensis* forests, using high-throughput sequencing to characterize bacterial and fungal communities and their links to soil stoichiometry. We propose the following hypotheses: (1) Natural forest rhizosphere soils support higher nutrient availability and fertility, sustained by specific beneficial microbial consortia. (2) The managed environment of ex situ plantations selects for an alternative soil stoichiometric regime and microbial functional assembly, which follows a distinct developmental trajectory compared to natural forests. (3) These imbalances can be diagnostically linked to key shifts in microbial community structure and function.

## 2. Results

### 2.1. Nutrient Content and Its Ecological Stoichiometric Ratio

Marked differences in rhizosphere soil physicochemical properties were observed across forest types and growth stages (Table 1). Soil pH, AP, NO_3_^−^-N and NH_4_^+^-N were generally lower in planted forests relative to natural forests at matched growth stages. SWC increased with tree size for both forest types. SOC and TN increased with growth stage in natural forests, while total phosphorus declined across growth stages in planted forests.

Two-way ANOVA (Table S1) revealed highly significant main effects of forest type, growth stage, and their forest type × growth stage interaction on pH, SWC, SOC, TN, TP, AP and NH_4_^+^-N (*p* < 0.001). For NO_3_^−^-N, growth stage and the interaction term were significant (*p* < 0.001), but the main effect of forest type was not significant (*p* = 0.0370). Partial omega-squared (ω^2^P) indicated that growth stage explained the largest proportion of variance for pH, TN and NH_4_^+^-N, while the interaction between forest type and growth stage also contributed substantially to the variation in these variables (Table S1).

**Figure 1:**
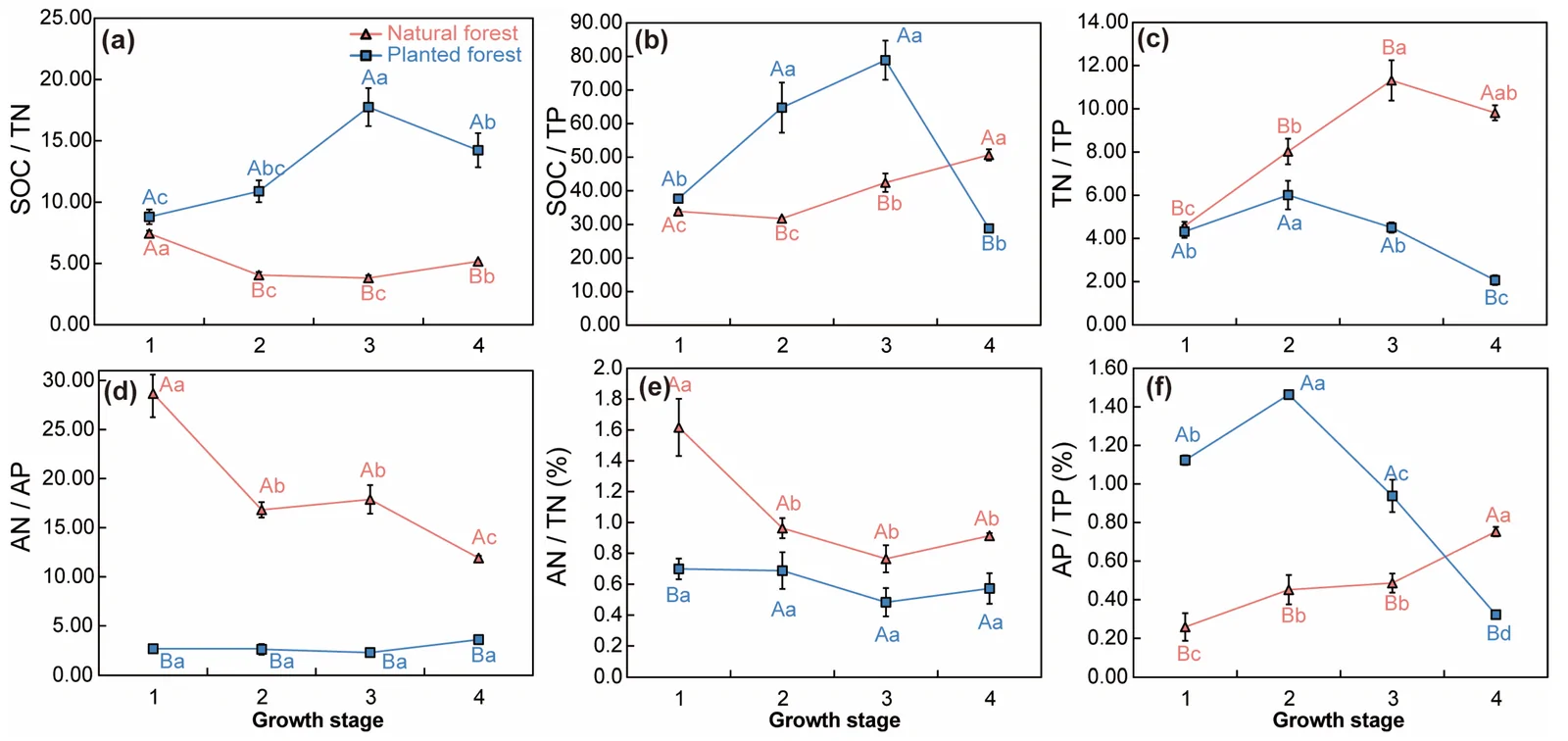
Soil nutrients and their ecological stoichiometry ratios. (**a**) SOC/TN ratio; (**b**) SOC/TP ratio; (**c**) TN/TP ratio; (**d**) AN/AP ratio; (**e**) AN/TN ratio; (**f**) AP/TP ratio. Different uppercase letters indicate significant differences between natural forests and planted forests (*p* < 0.05), and different lowercase letters indicate significant differences in different growth stages (*p* < 0.05) (*n* = 3).

The soil nutrient ratios of TN/TP, AN/AP, and AN/TN were consistently higher in NF than in PF across all growth stages. For the first three growth stages, SOC/TN, SOC/TP and AP/TP ratios were significantly higher in PF relative to NF (*p* < 0.05). These stoichiometric ratios generally showed an increasing trend in NF; by contrast, in PF the ratios rose initially and subsequently declined, with values converging toward those observed in NF at later growth stages. These observations show that NF exhibited higher N/P ratios and greater N availability, while PF was characterized by higher SOC/TN ratios and relatively elevated P availability. Furthermore, N and P availability metrics in PF became more similar to those in NF at later growth stages (Figure 1).

### 2.2. Microbial Biomass, Enzyme Activity, and Their Stoichiometry

**Figure 2:**
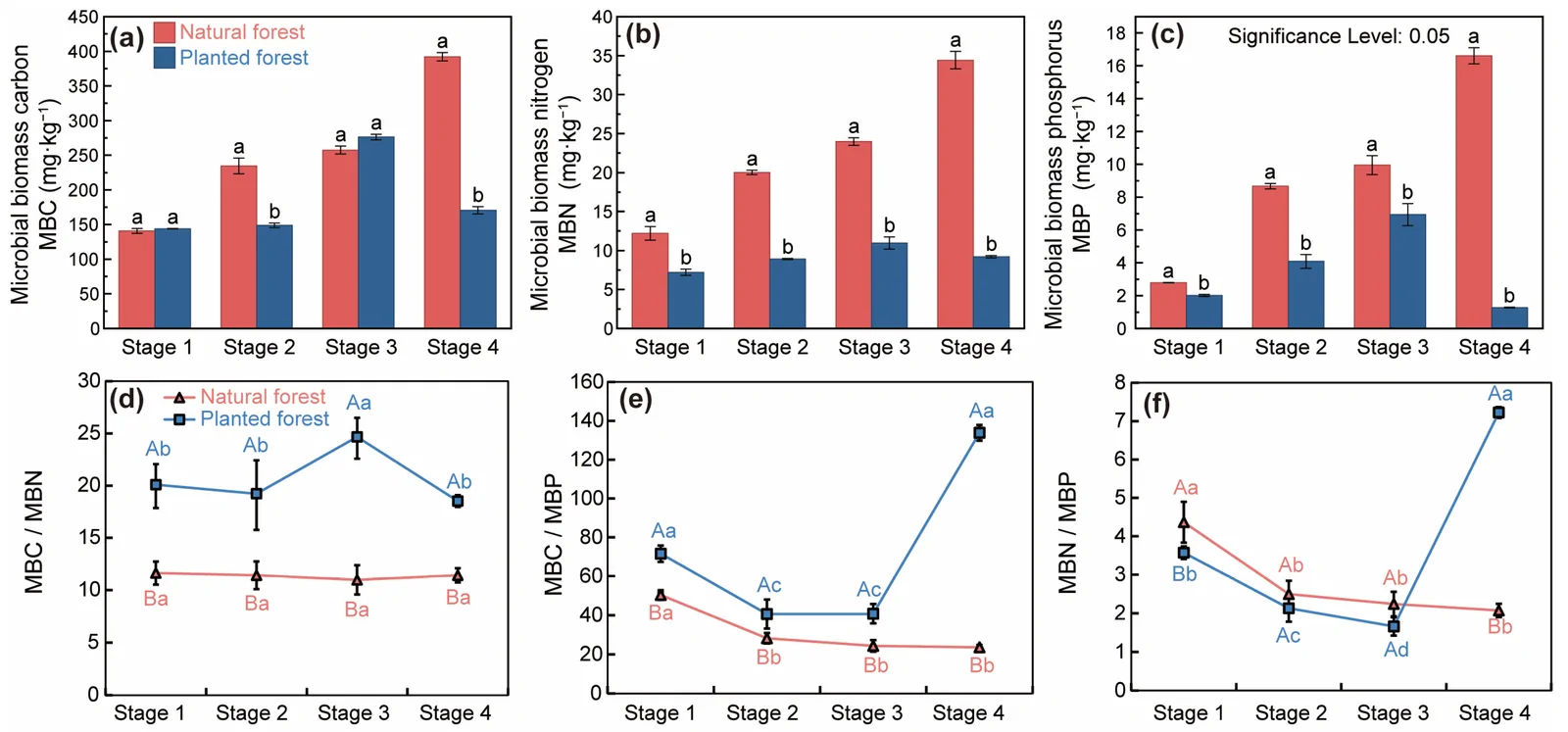
Microbial biomass of rhizosphere soils and its ecological stoichiometry ratios. (**a**) Microbial biomass carbon (MBC); (**b**) Microbial biomass nitrogen (MBN); (**c**) Microbial biomass phosphorus (MBP); (**d**) MBC/MBN ratio; (**e**) MBC/MBP ratio; (**f**) MBN/MBP ratio. Different uppercase letters indicate significant differences between natural forests and planted forests (*p* < 0.05), and different lowercase letters indicate significant differences in different growth stages (*p* < 0.05) (*n* = 3).

Soil microbial biomass carbon (MBC), microbial biomass nitrogen (MBN), and microbial biomass phosphorus (MBP) were significantly higher in NF than in PF. These variables varied significantly across tree developmental stages, showing an increasing trend along the developmental gradient for both forest types, but declined at the fourth developmental stage in PF (Figure 2a–c; Table S2). The MBC/MBN and MBC/MBP ratios were markedly higher in PF relative to NF, and the MBN/MBP ratio in PF exceeded that of NF significantly at the fourth developmental stage (Figure 2d–f).

**Figure 3:**
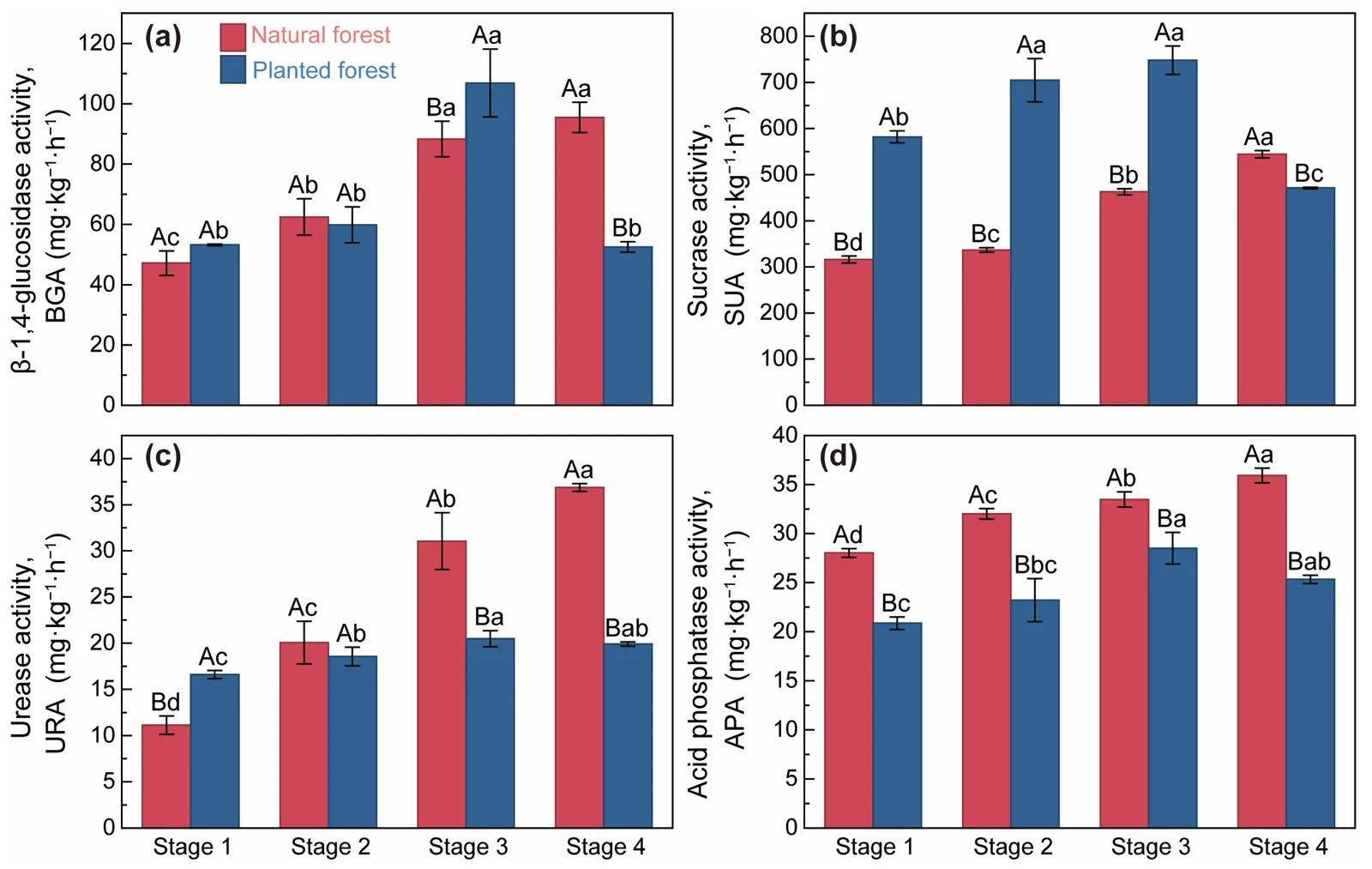
Extracellular enzyme activities of rhizosphere soils. (**a**) β-1,4-glucosidase (BGA); (**b**) Sucrase (SUA); (**c**) Urease (URA); (**d**) Acid phosphatase (APA). Different uppercase letters indicate significant differences between natural forests and planted forests (*p* < 0.05), and different lowercase letters indicate significant differences in different growth stages (*p* < 0.05) (*n* = 3).

In the first developmental stage, sucrase (SUA) and urease (URA) activities were significantly higher in PF than in NF (*p* < 0.05), while β-1,4-glucosidase (BGA) activity was also greater in PF. By contrast, acid phosphatase (APA) activity was consistently higher in NF across all developmental stages. All four extracellular enzyme activities exhibited significant variation across developmental stages (Table S3). Enzyme activities generally increased along the developmental gradient, with a reduction observed at the fourth stage in PF (Figure 3). In early developmental stages, SUA, URA, and BGA activities in PF exceeded those of NF; however, these enzyme activities decreased in PF at later developmental stages, while corresponding values in NF remained stable or increased, widening the difference between forest types.

**Figure 4:**
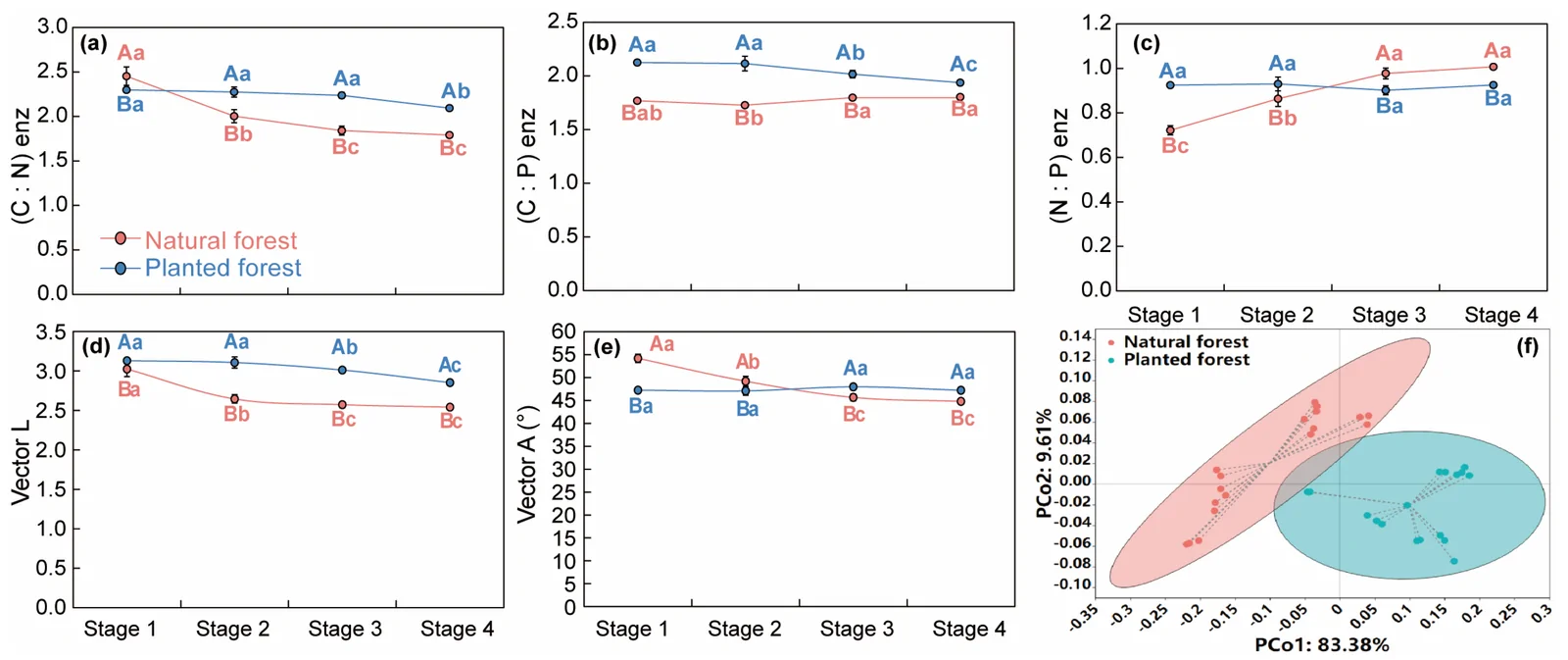
Stoichiometry ratios and principal component analysis of rhizosphere soil enzymes. (**a**) Enzymatic C:N ratio; (**b**) Enzymatic C:P ratio; (**c**) Enzymatic N:P ratio; (**d**) Vector length (Vector L); (**e**) Vector angle (Vector A); (**f**) Principal coordinate analysis (PCoA) of rhizosphere soil enzyme stoichiometry. Different uppercase letters indicate significant differences between natural forests and planted forests (*p* < 0.05), and different lowercase letters indicate significant differences in different growth stages (*p* < 0.05) (*n* = 3).

For most developmental stages, enzyme-based C:N stoichiometry (C:N)enz and enzyme-based C:P stoichiometry (C:P)enz were significantly higher in PF than in NF. The enzyme-based N:P stoichiometry (N:P)enz increased along the developmental gradient in NF and exceeded values of PF in the latter two developmental stages. Vector L was consistently higher in PF, whereas Vector A gradually increased in PF and surpassed NF in the later two developmental stages. Along the tree developmental gradient, the magnitude of differences in these enzyme stoichiometric indices between PF and NF diminished (Figure 4a–e). Principal coordinate analysis further confirmed clear separation of rhizosphere soil enzyme stoichiometric profiles between NF and PF (Figure 4f). Collectively, although rhizosphere soil enzyme stoichiometry differed substantially between ex situ conservation plantations and natural forests, the magnitude of inter-group differences decreased at later tree developmental stages along the sampled DBH gradient. Two-way ANOVA showed that forest type, tree developmental stage, and their interaction all significantly affected microbial biomass and soil enzyme indicators (Tables S2 and S3).

### 2.3. Microbial Quotient and Stoichiometric Imbalance

The qMBC, qMBN and qMBP were significantly higher in natural forests (NF) than in ex situ conservation plantations (PF). Along the tree developmental gradient, PF showed rising qMBC and qMBN in later stages, decreasing the magnitude of differences from NF (Table 2). Two-way ANOVA confirmed significant effects of forest type, growth stage, and their interaction on microbial quotients and stoichiometric imbalance (Table S4).

The (C/N)imb of PF increased continuously and exceeded NF in mid-to-late growth stages. However, PF (C/P)imb and (N/P)imb decreased sharply at the fourth stage and fell below NF values (Table 2). Rhizosphere microbial nutrient imbalance differed markedly between forest types along the developmental gradient, with divergent shifts in C-P and N-P imbalance in PF at later growth stages.

### 2.4. Microbial Community Diversity in Rhizosphere Soil

**Figure 5:**
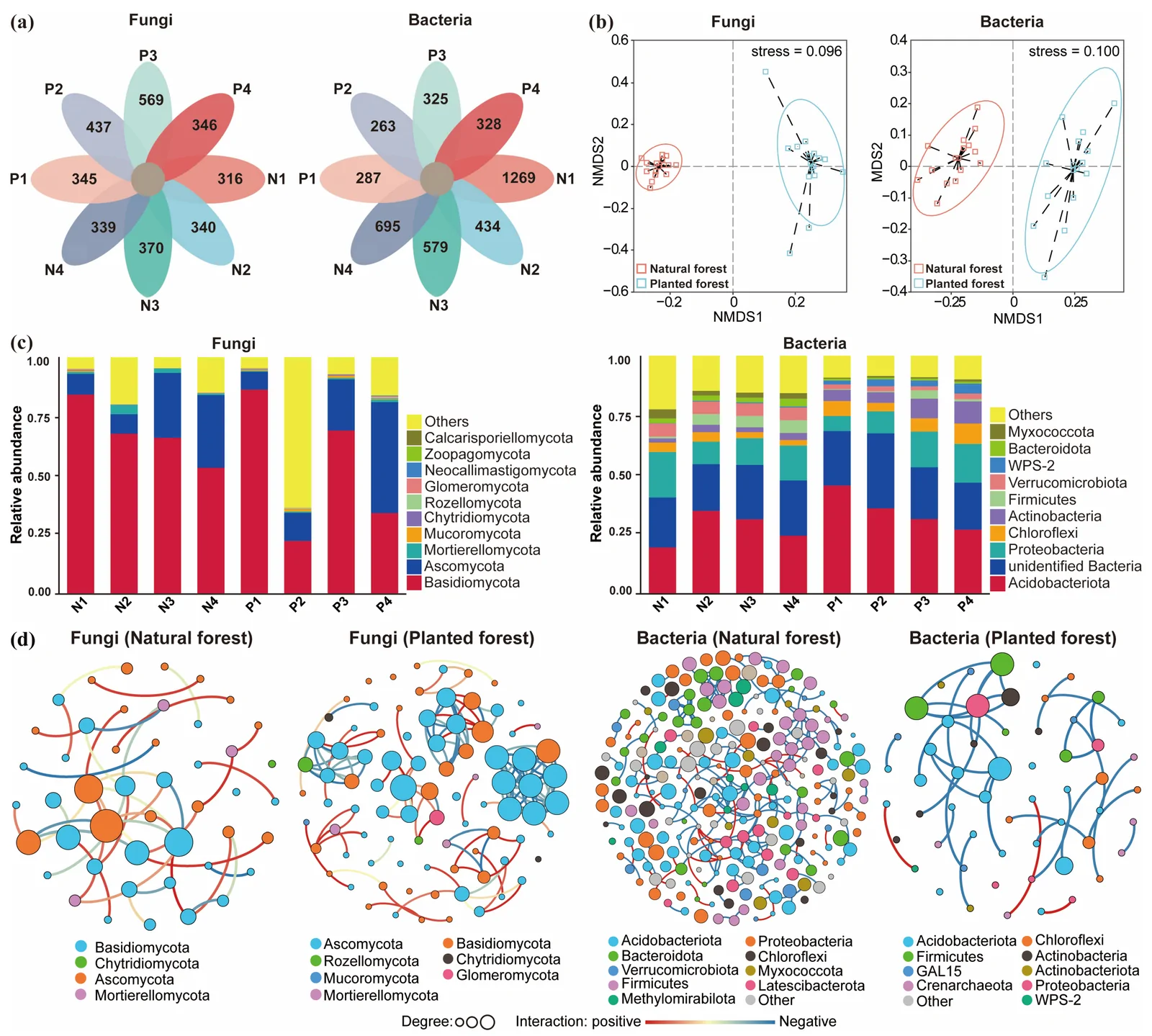
Community structure and diversity of rhizosphere soil microorganisms and their co-occurrence network diagram. Note: (**a**) Venn diagrams showing the numbers of shared and unique OTUs (*n* = 3); (**b**) non-metric multidimensional scaling (NMDS) ordination based on Bray–Curtis distance illustrating the β-diversity patterns; (**c**) stacked bar charts displaying the relative abundance of microbial taxa at the phylum level; and (**d**) microbial co-occurrence networks at the phylum level. In subplot (**d**), node size represents node degree, and red and blue edges indicate positive and negative interactions, respectively.

The number of operational taxonomic units (OTUs) of fungi was consistently higher in planted forests (PF) than in natural forests (NF), and bacteria were higher in NF. As trees grew, the number of fungal OTUs exhibited a pattern of initially increasing and then decreasing, and the bacteria showed the opposite trend (Figure 5a). The results of non-metric multidimensional scaling (NMDS) analysis indicated significant differences in the community structure diversity of bacteria and fungi between PF and NF (Figure 5b). The fungal phyla Basidiomycota and Ascomycota, as well as the bacterial phyla Acidobacteriota and Proteobacteria, were the dominant communities. However, the relative abundances of Basidiomycota and Acidobacteriota both showed an overall decreasing trend with tree growth, which was particularly more pronounced in PF (Figure 5c). The co-occurrence network analysis of microbial communities in NF and PF revealed that phyla with relatively high abundances, such as Basidiomycota, Ascomycota, Acidobacteriota, Proteobacteria, Bacteroidota, Firmicutes, Chloroflexi, and Actinobacteria, belonged to the dominant “co-varying groups”. They exhibited a strong tendency of “concurrent increase or decrease” in abundance, reflecting a closely interconnected network relationship, especially the bacterial community in NF (Figure 5d).

**Figure 6:**
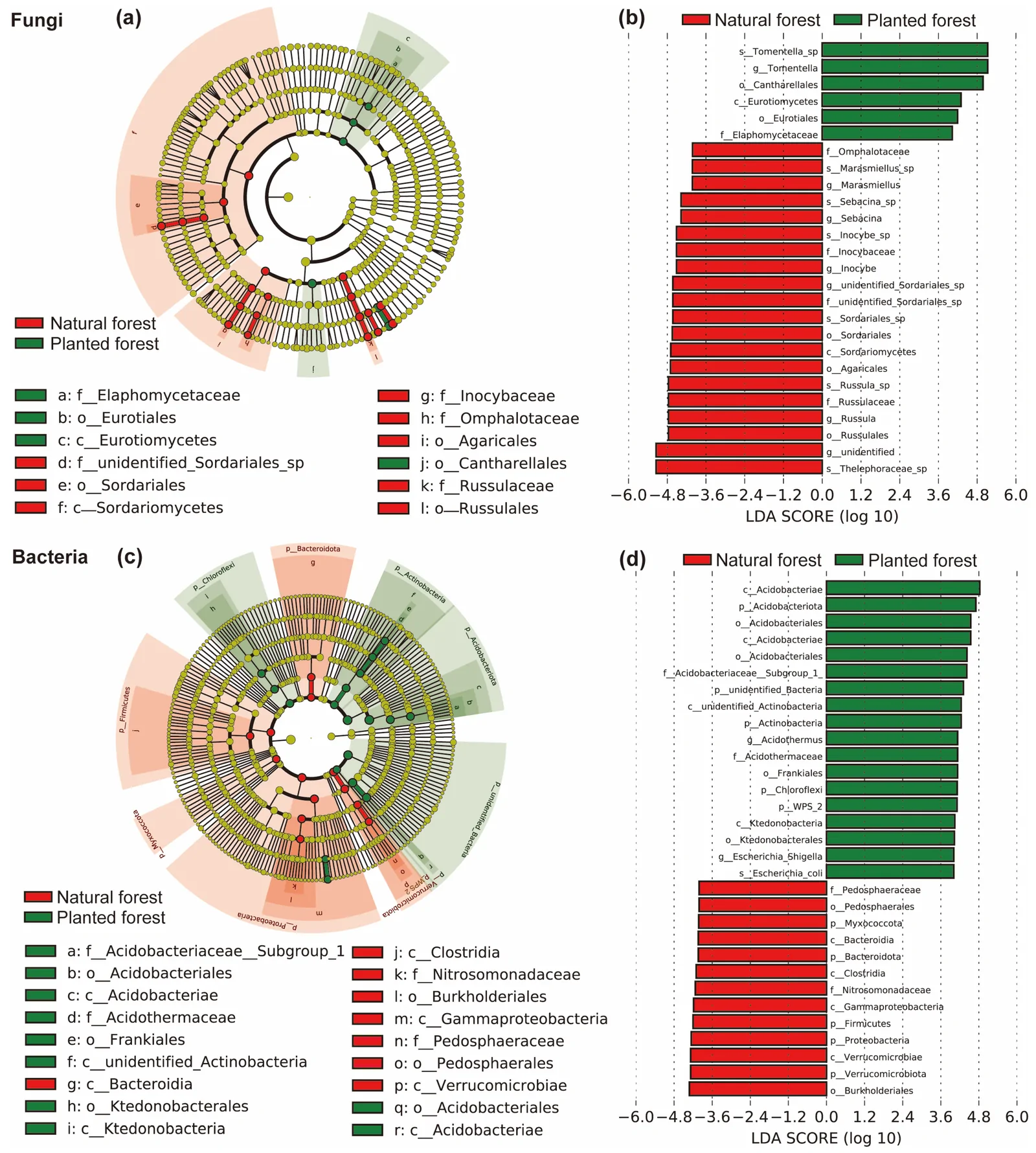
Differential fungal and bacterial taxa between natural forests and ex situ conservation plantations. Note: (**a**,**c**) LEfSe evolutionary cladograms for fungi and bacteria; (**b**,**d**) histograms showing linear discriminant analysis (LDA) scores, with the LDA threshold set to 4. Red nodes denote taxa enriched in natural forests, while green nodes denote taxa enriched in plantations (*n* = 3).

The LDA effect size cladogram revealed significant differences in microbial taxa with abundance variations between rhizosphere soils of *P. chinensis* NF and PF (Figure 6a,c). NF exhibited 20 fungal biomarkers playing crucial ecological roles, compared to only six in PF. Conversely, PF showed 18 bacterial biomarkers, while NF had 13 (Figure 6b,d). The fungal key taxa are more abundant in NF, while the bacterial key taxa are more abundant in PF.

**Figure 7:**
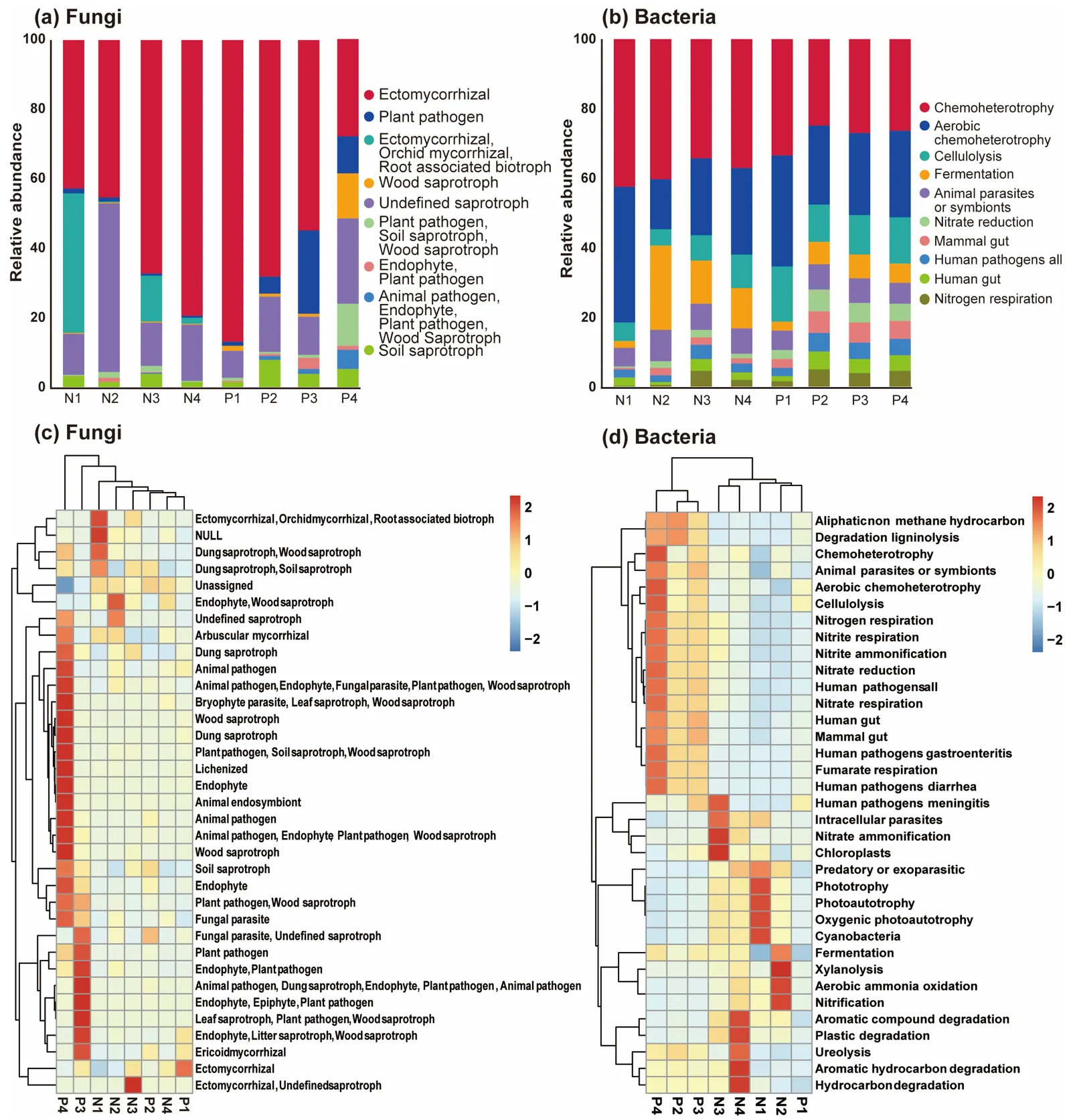
Predicted functional profiles of fungal and bacterial communities in natural forests and ex situ conservation plantations. Note: (**a**,**b**) Stacked bar charts showing the relative abundance of fungal and bacterial functional groups, respectively; (**c**,**d**) heatmaps illustrating the normalized relative abundance of predicted functional groups for fungi and bacteria across samples. The color gradient represents normalized abundance values (*n* = 3).

Prediction of microbial ecological functions showed that ectomycorrhizal fungi dominated the fungal community. The relative abundance of ectomycorrhizal fungi in natural forests (NF) rose continuously from 13.4% to 42.0% along the developmental gradient, accompanied by reduced fungal functional richness (Figure 7a). By contrast, both fungal functional richness and evenness increased in ex situ conservation plantations (PF). Dominant bacteria consistently had higher functional richness and evenness in PF than in NF across all developmental stages, with both indices increasing with tree development (Figure 7b). Functional clustering revealed higher functional heat values for fungal and bacterial communities in early developmental stages (N1, N2) of NF, indicating greater functional diversity, followed by declines in later stages (N3, N4). In PF, functional heat values increased markedly in later stages (P3, P4) (Figure 7c). Collectively, microbial functional diversity decreased with tree development in NF, while PF exhibited higher microbial functional diversity at later developmental stages (Figure 7d).

### 2.5. Relationship Between Soil Microbial Diversity and Soil Ecological Stoichiometry

**Figure 8:**
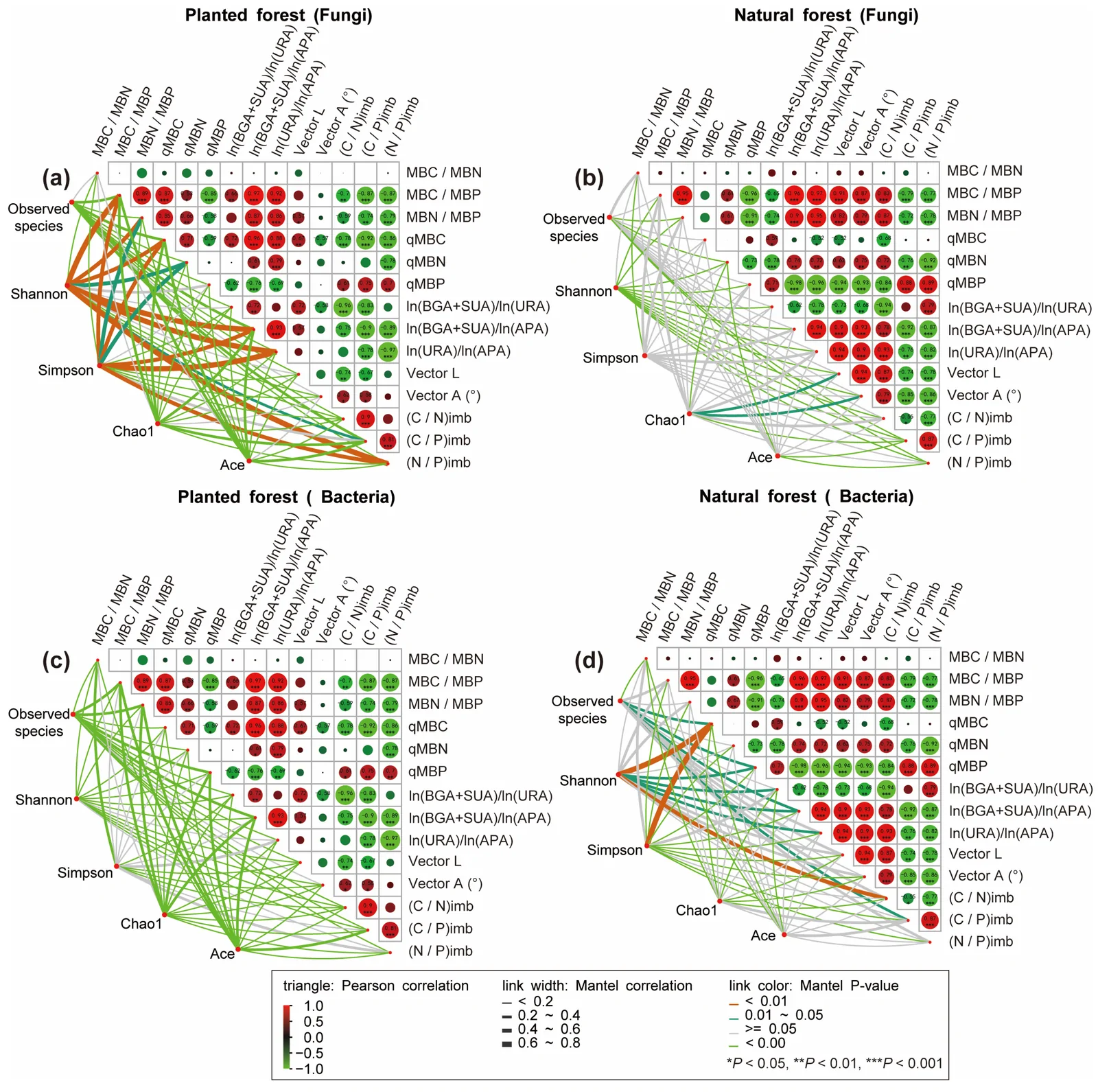
Mantel test showing correlations between microbial diversity indices and soil stoichiometric imbalance indicators across natural forests and ex situ conservation plantations. Note: Triangle color represents Pearson correlation coefficient. Link width indicates Mantel correlation coefficient (r), and link color denotes the *p*-value of the Mantel test (*n* = 3).

Mantel correlation analysis between rhizosphere microbial α-diversity indices and stoichiometric properties and stoichiometric imbalance revealed significant Mantel correlations (*p* < 0.05 or 0.01) between fungal Shannon and Simpson indices and multiple stoichiometric variables (MBC/MBP, MBN/MBP, qMBC, qMBN, ln(BGA+SUA)/ln(URA), ln(BGA+SUA)/ln(APA), ln(URA)/ln(APA), (C/P)imb, (N/P)imb) in PF (Figure 8a,c). In NF, bacterial observed species, Shannon, and Simpson indices were significantly correlated with qMBC, qMBP, ln(BGA+SUA)/ln(URA), ln(BGA+SUA)/ln(APA), ln(URA)/ln(APA), (C/N)imb and (C/P)imb (Figure 8b,d).

Two-way PERMANOVA showed that forest type, developmental stage, and their interaction significantly structured fungal and bacterial community composition (Table S5). PERMDISP verified significant multivariate dispersion differences for both microbial groups (Table S6). These results revealed divergent coupling relationships between microbial α-diversity and stoichiometric metrics: fungal diversity in PF was more strongly associated with stoichiometric ratios and imbalance, whereas bacterial diversity in NF was closely coupled to these soil nutrient indices.

### 2.6. Effects of Rhizosphere Soil Microorganisms on Nutrient Stoichiometry and Availability

**Figure 9:**
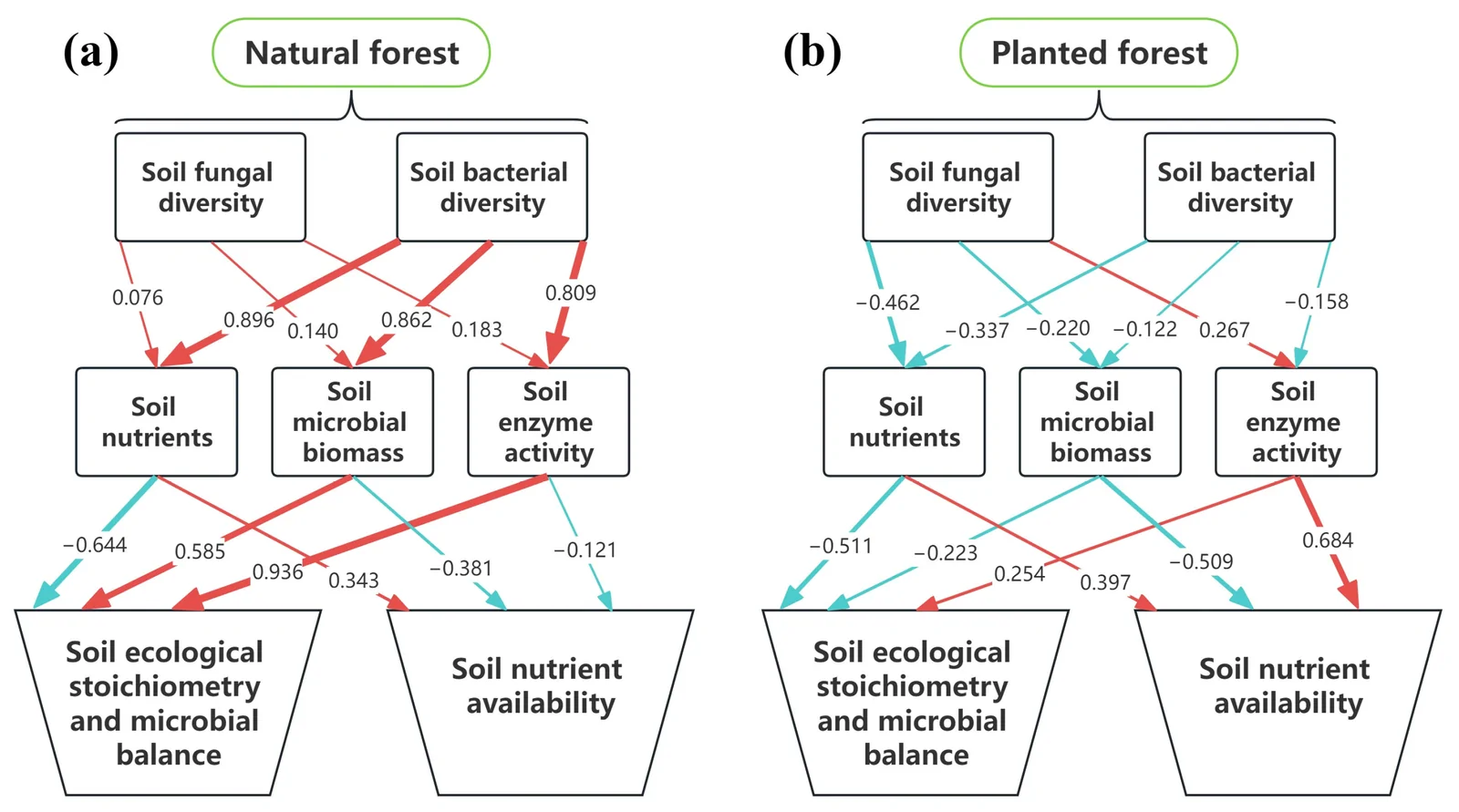
Structural equation modeling (SEM) reveals the causal pathways linking soil microbial diversity to soil nutrient stoichiometric balance and nutrient availability across natural forests and ex situ conservation plantations. (**a**) SEM for natural forest; (**b**) SEM for ex situ conservation plantation. Values beside arrows denote standardized path coefficients. Red arrows represent positive effects, and cyan arrows represent negative effects.

PLS-SEM indicated distinct correlative association patterns of rhizosphere microorganisms between the two forest types. In NF, rhizosphere soil microorganisms, particularly bacteria, showed positive associations with soil nutrient content, microbial biomass, and enzyme activities, which were correlated with more-balanced soil ecological stoichiometry (Figure 9a). In PF, fungi exhibited positive associations with soil enzyme activities; higher enzyme activity was further correlated with greater soil nutrient availability and reduced ecological stoichiometric imbalance (Figure 9b).

## 3. Discussion

### 3.1. Changes in Ecological Stoichiometric Ratio in Rhizosphere Soil

This study documents a distinct dynamic in the soil stoichiometry of *P. chinensis* ex situ plantations compared to natural forests. The overall trend indicates a partial recovery in the concentrations of certain nutrient pools over time [17]. However, this recovery is accompanied by a persistent and marked reduction in the soil N/P ratio across all developmental stages, together with a pronounced and progressive acidification process. This coexistence of apparent nutrient accumulation and fundamental stoichiometric shift underscores a functional divergence in plantation ecosystems, reflecting the complex outcomes of managed restoration [18]. The underlying mechanism for this persistent stoichiometric imbalance appears to be rooted in the initial management interventions and subsequent alterations to biogeochemical cycling.

The recovery of individual soil nutrient pools was asynchronous and unstable in PF. Consistent with our results (Table 1), TN, TP and NH_4_^+^-N in young PF (P1) were markedly lower than those in NF at the matched growth stage (*p* < 0.05), reflecting incomplete nutrient cycling in young plantation stands [17]. With stand development, SOC and AP accumulated rapidly in PF from P2 to P3 and even exceeded the corresponding values in NF. This demonstrates that tree growth and litter inputs can promote the replenishment of specific soil nutrient pools [19]. However, AP exhibited a nonlinear trajectory, peaking at intermediate growth stages and declining thereafter. Such asynchronous dynamics between nitrogen and phosphorus pools are the primary driver of the persistently low N/P stoichiometry in PF throughout stand development. The chronically low N/P environment in PF creates a nutrient supply regime that differs substantially from the native soil conditions of *P. chinensis* in natural forests [5,20]. Therefore, the plantation soil system is not simply nutrient-depleted; instead, it establishes a distinct edaphic environment with rebalanced nutrient stoichiometry. This altered soil environment may exert strong selection pressure on plant nutrient-acquisition strategies and further reshape the composition, diversity and function of rhizosphere microbial communities [21].

Moreover, progressive soil acidification interacts synergistically with nutrient stoichiometric imbalance to create compound edaphic stress for root and microbial processes [22]. Soil pH declined substantially across growth stages and was consistently lower in PF than in NF. Under strongly acidic conditions, phosphorus is readily fixed by reactive Fe and Al oxides, which greatly reduces the bioavailability of soil phosphorus [23]. Accordingly, even when the measurable pool of AP appears relatively high at intermediate growth stages, its actual bioavailability for plants and rhizosphere microbes remains constrained. The combination of low N/P stoichiometry and severe soil acidification exacerbates phosphorus limitation in PF, forming a composite edaphic stressor driven by the coupling of stoichiometric imbalance and soil acidification [1]. Characterizing this integrated soil condition provides a critical foundation for interpreting belowground ecosystem processes, evaluating the long-term success of *P. chinensis* ex situ conservation plantations, and developing targeted soil management practices to mitigate these coupled nutrient limitations.

### 3.2. Impact of Management Practices on Soil Microbial Diversity

Our findings show that management practices fundamentally reorganize rhizosphere microbial communities, creating a system functionally different from natural forests [24]. Contrary to the bacterial-dominated NF, the PF exhibited a fungal-centric assembly. Specifically, fungal OTUs in the rhizosphere soil of PF of *P. Chinensis* were consistently higher in the PF across all growth stages, showing an initial increase, while bacteria level was generally lower and exhibited a contrasting temporal trend (Figure 6a). This pattern, where forest restoration or management alters nutrient availability and selects for distinct microbial communities, has been observed in similar ecosystems [25]. Two-way PERMANOVA and PERMDISP further confirmed significant divergence in fungal and bacterial community composition between NF and PF, accompanied by significant differences in multivariate dispersion (Tables S5 and S6). This community separation reflected fundamental shifts in community composition rather than simple changes in microbial abundance.

In line with the characteristic soil microbiome of karst regions in southwestern China, where Proteobacteria, Acidobacteria, and Actinobacteria are established as dominant bacterial phyla [26], our results further delineate that Ascomycota was the major fungal group in the soil of natural and planted forests [27]. Critically, functional implications are profound. In the NF, the increasing abundance of ectomycorrhizal fungi with stand age (Figure 8a) aligns with its higher N/P regime, facilitating efficient nutrient acquisition in a more-balanced stoichiometric environment [28]. In stark contrast, the relative abundance of ectomycorrhizal fungi in PF remained low and showed no consistent increasing trend (Figure 7a). Instead, the PF fostered a microbial community with higher overall fungal functional richness and evenness (Figure 7a,b). This likely represents a compensatory functional redundancy, a response to the combined stresses of stoichiometric imbalance and acidification. Through this reorganization, the managed system develops a complex microbial portfolio that may enhance stability and resource utilization under chronic abiotic stress [29]. Furthermore, Venn diagrams and NMDS analysis indicate significant differences in fungal and bacterial communities between natural and planted forests. These differences may be driven by groups such as Proteobacteria and Basidiomycota [30]. The co-occurrence network analysis further revealed that main phyla, including Basidiomycota, Ascomycota, Proteobacteria and Acidobacteriota formed strongly correlated co-varying groups, with tight interspecific associations, especially for bacterial communities in NF (Figure 5d).

Our findings highlight that *P. chinensis* ex situ conservation plantations can reconstruct and sustain unique rhizosphere microbial functional profiles. Artificial management modulates soil microhabitat conditions and provides favorable environments for microbial colonization and community assembly [31]. Increased microbial functional diversity can promote soil fertility and sustain key belowground ecosystem services, which is beneficial for the long-term success of ex situ conservation [32]. Although natural secondary forest regeneration is reported to develop microbial communities more similar to primary forests and better restore soil microbial structure and function [33], our study indicates that ex situ conservation plantations do not merely alter microbial species composition. On the contrary, management practices have led to the formation of a new, primarily fungal microbial community [30]. This fungus-centered assemblage in plantations relies on alternative biogeochemical pathways to maintain soil processes. Such functional divergence must be fully considered when evaluating the long-term viability and ecological equivalence of *P. chinensis* conservation plantations, because the belowground microbial functional pathways in PF cannot be directly equated to those observed in the native natural forest ecosystem [32].

### 3.3. Effect of Ex Situ Conservation on the Relationship Between Stoichiometry and Microbial Communities

Ex situ conservation markedly reshapes the coupling relationships between soil stoichiometry and rhizosphere microbial communities of *P. chinensis*. Artificial management creates an edaphic environment with progressive acidification and persistently low N/P ratios, where phosphorus constitutes the primary limiting nutrient in these karst soils [34]. Mantel tests revealed divergent associations: fungal α-diversity was tightly coupled with stoichiometric imbalance in PF, while bacterial diversity dominated such correlations in NF. The combined stress of low N/P stoichiometry and low pH imposes persistent phosphorus constraints on rhizosphere microbes, which may partly stem from differences in litter inputs between the two forest types [35].

Core microbial metabolism retains partial resilience under nutrient limitation. Significant positive correlations between C-, N-, and P-acquiring enzyme activities and microbial biomass were maintained in both forest types [36], yet this resilience has clear boundaries, reflected by consistently low AP/TP ratios across all growth stages in PF [37]. Lower microbial quotients (qMBC, qMBN, qMBP) in PF indicate reduced microbial resource-use efficiency relative to NF.

The stoichiometric conditions in conservation plantations drive specific microbial adaptations. The significantly higher (C/N)imb in planted forests reflects relative N limitation, promoting the development of fungal-dominated communities that are better equipped to utilize resources in such environments [38]. Stronger microbe stoichiometry correlations for fungi in PF and bacteria in NF support this differentiation. Declining (C/P)imb and (N/P)imb in later growth stages represent microbial physiological responses to P limitation [39]. PLS-SEM further revealed divergent regulatory pathways: bacteria mediated nutrient cycling and stoichiometric balance in NF, whereas fungi regulated enzyme activity and alleviated stoichiometric imbalance in PF.

These findings demonstrate that successful ex situ conservation must consider belowground biogeochemical processes rather than merely plant survival [40]. We propose inoculation with native key microbial taxa (ectomycorrhizal fungi and P-solubilizing bacteria) from natural forests to mitigate stoichiometric imbalance, improve nutrient cycling efficiency [41,42], and sustain the long-term conservation of this endangered dipterocarp species.

### 3.4. Ecological Implications for Conservation Practice

This observational study showed that the rhizosphere ecological characteristics of *P. chinensis* ex situ conservation plantations differed markedly from those of natural forests and did not replicate the belowground properties of natural forests. The plantations exhibited unique soil and microbial features, representing adaptive traits of *P. chinensis* under the ex situ artificial habitat. Along the DBH gradient, key soil nutrient cycling indicators and microbial community attributes of plantations gradually approached those of natural forests with tree development. These findings provided insights for evaluating ex situ conservation of endangered tree species: the assessment criterion for ex situ conservation did not require belowground ecosystems to fully match natural forests; instead, priority was given to evaluating ecosystem functional integrity and developmental potential.

Notably, natural and plantation stands were located at separate sites with distinct topography, understory vegetation, and management practices. Thus, the observed differences in soil stoichiometry and microbial communities could not be simply attributed to ex situ afforestation and tending. Moreover, differences observed across DBH classes could not be directly interpreted as long-term temporal ecological succession. The stage-dependent shifts in nutrients and microbes observed in this study only reflected features under the current site conditions. Further paired-site surveys, manipulative experiments, and long-term monitoring are needed to disentangle the independent effects of topography, site conditions, and forest management, and to assess the long-term sustainability of the soil–microbe system in ex situ *P. chinensis* plantations.

## 4. Materials and Methods

### 4.1. Experimental Site

**Figure 10:**
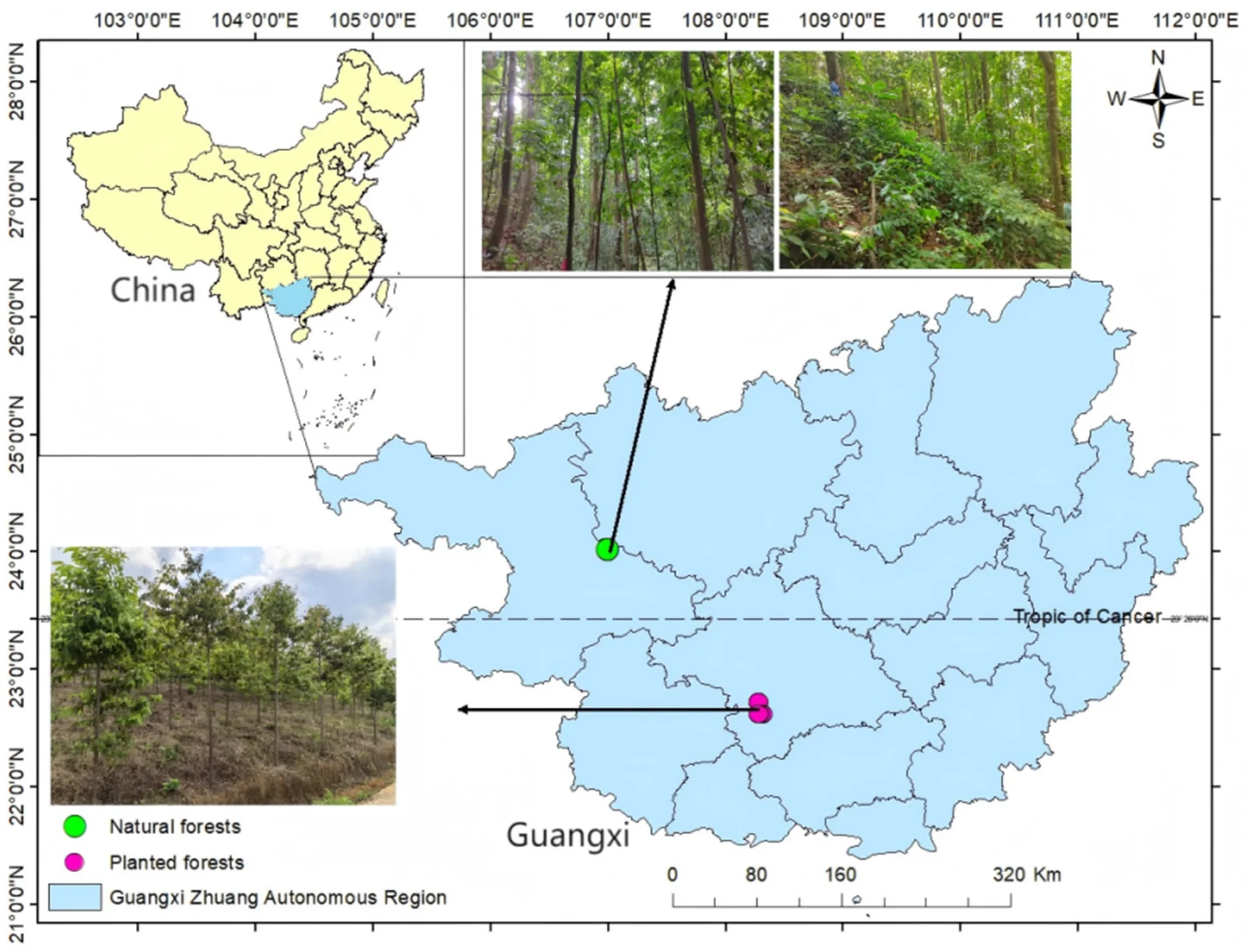
Geographical location of natural forests and planted forests plots of *Parashorea chinensis*.

This study focused on *Parashorea chinensis*. Two forest types, natural forest (NF) and ex situ conservation plantation forest (PF), were selected for investigation. The natural forest sites were located in the Youjiang Valley, Tianyang District, Baise City, Guangxi, with geographic coordinates of 24°1′24″ N, 106°59′38″ E and an elevation of approximately 416 m. The ex situ conservation plantation sites were situated in the suburbs of Nanning, with coordinates of 22°37′57″ N, 108°18′47″ E and an elevation of approximately 163 m. The study areas lie near the Tropic of Cancer within a karst region and feature a subtropical monsoon climate, with a mean annual precipitation of approximately 1300 mm and a mean annual temperature of around 20 °C (Figure 10).

The natural forest was an uneven-aged native stand, where *P. chinensis* accounted for 80% of all tree individuals. Its main associated tree species was *Ficus esquiroliana*. Understory vegetation included *Lespedeza bicolor*, *Rubus alceaefolius*, *Paederia foetida* and other herbaceous and shrubby species. The plantation was a pure stand of *P. chinensis*, established using seedlings propagated from seeds originating from the same seed source as the natural forest. Base fertilizer (0.25 kg per plant, N:P_2_O_5_:K_2_O = 15:6:9) was applied at the initial afforestation stage. Seeds of *P. chinensis* used for the ex situ plantation were harvested in 1978, 2011, 2012, 2017, 2018 and 2019. Seedlings underwent a 1–2-year nursery period prior to outplanting. Supplementary planting and regular understory clearing were conducted in subsequent years. Although the plantation was established in the same afforestation period, individual trees exhibited marked growth differentiation, forming an uneven-aged structure with multiple diameter-at-breast-height (DBH) classes within the stand. We compared the two forest types with identical seed sources and broadly similar climatic backgrounds to reduce confounding effects derived from seed source and regional climatic differences. Three independent natural forest stands (NF1, NF2, NF3) and three independent plantation stands (PF1, PF2, PF3) were established in this study. Each stand covered an area of 666 m^2^ (25.8 m × 25.8 m). The distance between any two natural forest stands was ≥500 m, and ≥800 m between plantation stands to ensure spatial independence. Each stand served as one biological replicate (*n* = 3). General information of the sampling stands is presented in Table 3.

### 4.2. Experimental Design and Soil Sampling Methodology

This study adopted a hierarchical experimental design: forest type—independent stand—growth stage—plot. Four growth stages were classified based on tree diameter at breast height (DBH) (Figure S1). As both forest types were uneven-aged stands, developmental stages could not be defined by stand age; thus, four growth stages of *P. chinensis* were delineated using individual DBH values [43,44]. Natural forest stages were designated N1–N4 and plantation stages P1–P4. Individuals of *P. chinensis* covering all four DBH classes were found within each independent stand. All trees in each stand were surveyed for DBH and assigned to one of four growth stages (Table 3).

Within each growth stage, five healthy target trees were selected for rhizosphere soil sampling. Rhizosphere soil adhering to root surfaces (0–2 mm) was collected via the shaking method combined with sterile brushes. Rhizosphere soils from the five trees within the same growth stage were composited into a single biological sample. Ultimately, three biological replicates (*n* = 3) were obtained for each growth stage for analyses of soil physicochemical properties, enzyme activities, microbial biomass and high-throughput sequencing. Each composite sample was divided into three subsamples: one air-dried, ground and sieved for soil physicochemical and nutrient analyses; one stored fresh at 4 °C for enzyme activity and microbial biomass phosphorus assays; and the third transported in liquid nitrogen and preserved at −80 °C in the laboratory for high-throughput sequencing of soil bacterial and fungal communities.

### 4.3. Determination of Soil Physicochemical Properties and Nutrient Content

Air-dried soil samples were sieved through a 0.15 mm mesh. Soil pH was determined using a pH meter (Model PHBJ-260, Shanghai INESA Scientific Instrument Co., Ltd., Shanghai, China) following a 1:2.5 (*w/v*) soil:water extraction method. Soil water content (SWC) was determined by the weighing method; soil organic carbon (SOC) was determined by the potassium dichromate-concentrated sulphuric acid (K_2_Cr_2_O_7_-H_2_SO_4_, Sinopharm Chemical Reagent Co., Ltd., Shanghai, China) plus thermal oxidation colorimetric method; total nitrogen (TN) was determined by sulphuric acid-catalyst ablation method; and the contents of total phosphorus (TP) and total potassium (TK) were determined by the NaOH melting method and inductively coupled plasma mass spectrometry (ICP-MS, Model 7900, Agilent Technologies, Inc., Santa Clara, CA, USA) [45]. Soil available phosphorus (AP) was determined by double acid (0.05 mol·L^−1^ HCl, and 0.025 mol·L^−1^ (1/2H_2_SO_4_)) leaching and filtration method; available potassium (AK) was determined by flame photometer (model FP6410, INESA Scientific Instrument Co., Ltd., Shanghai, China); ammonium N (NH_4_^+^-N) and nitrate N (NO_3_^−^-N) were determined by KCl leaching method, and available nitrogen (AN) was calculated as the sum of NH_4_^+^-N and NO_3_^−^-N contents.

### 4.4. Determination of Soil Microbial Biomass and Enzyme Activity

Soil microbial biomass carbon (MBC), nitrogen (MBN), and phosphorus (MBP) were determined using the chloroform fumigation-extraction method. Briefly, fresh soil samples were fumigated with ethanol-free chloroform in the dark at 25 °C for 24 h. Fumigated and unfumigated paired soil samples were extracted with 0.5 mol L^−1^ K_2_SO_4_ for MBC and MBN, and with 0.5 mol L^−1^ NaHCO_3_ for MBP. MBC and MBN were calculated using a conversion coefficient Kc = 0.45, while MBP was calculated using Kp = 0.40 (Equations (1)–(3)). The unit was mg kg^−1^. Reagent blanks were set for each batch of extraction to eliminate background interference derived from solvents.

Soil β-1,4-glucosidase (BGA) activity was assayed using p-nitrophenyl-β-D-glucoside as the substrate. Samples were incubated at 37 °C for 1 h, and absorbance was measured at 410 nm using an ultraviolet visible spectrophotometer. Sucrase activity (SUA) was determined with sucrose as the substrate via the 3,5-dinitrosalicylic acid colorimetric method after incubation at 37 °C for 24 h, followed by color development. Urease activity (URA) was measured using urea as the substrate with the phenol sodium–sodium hypochlorite colorimetric method after incubation at 37 °C for 24 h. Acid phosphatase activity (APA) was assayed with disodium phenyl phosphate (C_6_H_9_Na_2_O_6_P) as the substrate; samples were incubated at 37 °C for 2 h and quantified by colorimetry. Substrate blanks and reagent blanks were prepared synchronously for each batch of colorimetric measurements of all enzymes. Enzyme activities were normalized to dry soil mass, with a unified unit of mg·kg^−1^ h^−1^. Units of enzyme activity from various categories are compatible and can be used for enzymatic stoichiometric calculations. The detailed measurement and calculation procedures followed Li et al. (2023) [46] (Equations (4)–(6)).

MBC = EC/Kc(1)

MBN = EN/Kc(2)

MBP = Ept/Kp(3)

EC, EN and Ept are the differences in organic carbon, total nitrogen, and total phosphorus in the leachate of fumigated and unfumigated soil samples, respectively.

(C:N)_enz_ = ln(BGA + SUA)/ln(URA) (4)

(C:P)_enz_ = ln(BGA + SUA)/ln(APA) (5)

(N:P)_enz_ = ln(URA)/ln(APA) (6)

### 4.5. High-Throughput Sequencing of Soil Microorganisms

Total genomic DNA was extracted from rhizosphere soil using the DNeasy PowerSoil Pro Kit (Qiagen, Germantown, MD, USA) following the manufacturer’s instructions. The DNA concentration and purity were determined with a NanoDrop 2000 spectrophotometer (Thermo Fisher Scientific, Waltham, MA, USA), and DNA integrity was checked via 1.0% agarose gel electrophoresis. Extraction blanks (no-soil controls) were included in each batch of DNA extraction. High-throughput sequencing for microbial communities of rhizosphere soil in this study was performed by Novogene Co., Ltd., Beijing, China. The V4 hypervariable region of the bacterial 16S rRNA gene was amplified using the primer pair 515F (5′-GTGCCAGCMGCCGCGGTAA-3′) and 806R (5′-GGACTACHVGGGTWTCTAAT-3′). For fungi, the ITS1 region was amplified with primers ITS5-1737F and ITS2-2043R. The TruSeq^®^ DNA PCR-Free Sample Preparation Kit was used to construct sequencing libraries. After library quality verification, paired-end sequencing was carried out on the Illumina NovaSeq 6000 platform (USA). No-template negative controls (NTC) were set for each PCR amplification run to detect contamination of PCR reagents.

Raw FASTQ sequencing reads were quality-filtered and assembled using QIIME2 (v2022.2). Reads with base quality scores < 20, ambiguous N bases, or lengths shorter than 200 bp were discarded. Paired-end reads were merged by VSEARCH, and chimeric sequences were removed using the uchime2 algorithm. Quality-filtered clean reads were clustered into OTUs at a 97% sequence similarity threshold. Taxonomic classification of representative OTU sequences was performed against the SILVA database (v138.1) for bacterial 16S sequences and the UNITE database (v8.3) for fungal ITS sequences. Only taxonomic assignments with a confidence threshold > 0.8 were retained. To reduce bias caused by uneven sequencing depth among samples, all samples were rarefied to the minimum sequencing depth.

### 4.6. Statistical Analyses

The formula for calculating soil extracellular enzyme stoichiometric ratios was optimized with reference to [47] (Equations (7) and (8)). Vector analysis of soil enzyme stoichiometry was used to characterize the limiting factors of soil nutrient cycling in order to quantify the ratio of soil enzyme activity to nutrient requirements [48,49]. The relative limiting quantities of C were expressed in terms of vector length (Vector L) and those of N and P were expressed in terms of vector angle (Vector A), calculated with reference to the equations mentioned by [46,50].

(7) $$\mathrm{Vector L}=\mathrm{SQRT}({\left({\left(C:N\right)}_{\mathrm{enz}}\right)}^{2}+{\left({\left(C:P\right)}_{\mathrm{enz}}\right)}^{2})$$

Vector A = DEGREES((ATAN2((C:P)_enz_); ln((C:N)_enz_))) (8)

Soil microbial quotient (qMBC, qMBN, qMBP) is the ratio of soil microbial biomass C, N and P to soil organic C, TN and TP, calculated with reference to the method mentioned by [46] (Equations (9)–(11)), and was used to reflect the amount of microbial biomass that the soil can support [51].

qMBC = MBC/SOC(9)

qMBN = MBN/TN(10)

qMBP = MBP/TP(11)

Soil microbial stoichiometric imbalances: (C/N)imb, (C/P)imb, (N/P)imb (Equations (12)–(14)) were calculated with reference to the method mentioned by [52].

(C:N)_imb_ = (SOC/TN)/(MBC/MBN) (12)

(C:P)_imb_ = (SOC/TP)/(MBC/MBP) (13)

(N:P)_imb_ = (TN/TP)/(MBN/MBP) (14)

In the statistical comparisons, the independent stand was the unit of replication, giving *n* = 3 stands per forest type for each growth stage. Microsoft Excel 2019 was used for data organization and calculation of all stoichiometric indices. IBM SPSS Statistics 25.0 was used to perform one-way ANOVA to test differences among growth stages, with significance thresholds set at *p* < 0.05 and *p* < 0.01. Two-way ANOVA was further applied to disentangle the main and interactive effects of forest type, growth stage, and their interaction on measured variables. Prior to ANOVA, model assumptions were verified for each response variable. The Shapiro–Wilk test was used to examine residual normality, and Levene’s test was applied to assess homogeneity of variances. Where residuals violated normality or homogeneity assumptions, data were log- or square-root-transformed. Variables that failed assumption checks after transformation were analyzed using the non-parametric Kruskal–Wallis test. Tukey’s HSD post hoc test was used for pairwise comparisons when ANOVA results were significant (*p* < 0.05).

For microbial community β-diversity (NMDS and PCoA), permutational multivariate analysis of variance (PERMANOVA, adonis2 function, 999 permutations, marginal sums of squares, by = “margin”) based on the Bray–Curtis distance matrix was performed to test the effects of forest type, growth stage and their interaction. Permutational analysis of multivariate dispersions (PERMDISP, betadisper with 999 permutations) was used to evaluate differences in multivariate dispersion among groups, to avoid confounding effects of within-group heterogeneity on PERMANOVA outcomes. Both NMDS and PCoA ordinations were constructed using the identical Bray–Curtis distance matrix. Taxonomic assignments were conducted across multiple taxonomic ranks; only annotations with confidence >0.8 were retained, and annotation coverage was calculated as the proportion of OTUs assigned to at least the kingdom level. Fungal and bacterial functional predictions were carried out using the FUNGuild and FAPROTAX databases, respectively [53]. All statistical analyses were conducted using R software (Version 4.3.1).

LEfSe software (Version 1.0) was used for linear discriminant analysis effect size (LEfSe) analysis. The non-parametric factorial Kruskal–Wallis sum-rank test was first applied to identify taxa with significantly different abundances between natural and plantation forests. A pairwise Wilcoxon rank-sum test was then used to assess between-group differences, followed by linear discriminant analysis (LDA) for dimension reduction and estimation of the effect size of discriminant taxa (LDA score), with the LDA threshold set to 4. OTUs with mean relative abundance <0.01% and occurrence in fewer than one-fifth of all samples were filtered in R. The WGCNA package was used to calculate network metrics and construct microbial co-occurrence networks, and Gephi 0.9.2 was used for network visualization [54].

## 5. Conclusions

This study examined rhizosphere soil physicochemical properties, nutrient stoichiometry, and bacterial and fungal communities of *Parashorea chinensis* across DBH-based developmental stages in natural forests and ex situ conservation plantations. Two-way ANOVA showed that forest type, developmental stage, and their interaction highly significantly affected soil pH, water content, organic carbon, total nitrogen, total phosphorus, available phosphorus, and ammonium nitrogen (*p* < 0.001). Growth stage dominated variations in most soil physicochemical, microbial biomass, microbial quotient, and stoichiometric imbalance indices, whereas their interaction controlled all enzyme activity metrics. Natural forest rhizosphere soils exhibited stronger nitrogen supply and relatively severe phosphorus limitation, while plantation soils had higher C:P ratios, greater relative P availability, and limited N supply. With tree development, rhizosphere stoichiometry in plantations gradually converged toward the regime of natural forests, suggesting that managed tree growth modulates rhizosphere nutrient ratios and reshapes nutrient limitation patterns. The forest type × growth stage interaction was the primary driver of fungal community variation, while bacterial community variation was governed only by the two main effects. This work provides field observational evidence for rhizosphere soil microbial shifts during *P. chinensis* ex situ conservation.

## Figures and Tables

**Table 1:** Physicochemical properties and nutrient content of rhizosphere soil.

Forest Stands	pH	Soil Water Content, SWC (%)	Soil Organic Carbon, SOC (g·kg^−1^)	Total Nitrogen, TN (g·kg^−1^)	Total Phosphorus, TP (g·kg^−1^)	Available Phosphorus, AP (mg·kg^−1^)	Nitrate Nitrogen, NO_3_^−^-N (mg·kg^−1^)	Ammonium Nitrogen, NH_4_^+^-N (mg·kg^−1^)
N1	5.96 ± 0.13 A a	20.29 ± 1.29 A b	11.98 ± 0.05 Ac	1.61 ± 0.10 A c	0.36 ± 0.01 A d	0.92 ± 0.08 B a	5.27 ± 0.18 A c	20.70 ± 1.54 A b
P1	4.84 ± 0.06 B a	8.66 ± 0.24 B c	11.98 ± 0.09 Ad	1.37 ± 0.16 A b	0.32 ± 0.01 B a	3.57 ± 0.19 A b c	2.93 ± 0.06 B c	6.61 ± 0.63 B a
N2	5.78 ± 0.07 A a	20.41 ± 0.44 A b	12.99 ± 0.12 Bc	2.81 ± 0.05 A b	0.40 ± 0.01 A b	1.85 ± 0.10 B b	5.40 ± 0.05 A c	25.93 ± 1.71 A a b
P2	4.52 ± 0.05 B b	9.47 ± 1.04 B c	16.65 ± 0.49 Ac	1.72 ± 0.37 B a b	0.35 ± 0.04 A a	4.33 ± 0.95 A b	3.56 ± 0.28 B c	6.43 ± 1.19 B a
N3	5.12 ± 0.05 A b	22.13 ± 0.10 A b	16.26 ± 0.91 Bb	4.12 ± 0.51 A a	0.44 ± 0.00 A a	2.15 ± 0.02 B a	6.73 ± 0.33 A b	26.64 ± 2.93 A a
P3	3.61 ± 0.11 B c	15.07 ± 1.46 B b	27.97 ± 0.43 Aa	2.43 ± 0.42 B a	0.24 ± 0.02 B b	6.71 ± 0.24 A a	5.16 ± 0.21 B b	5.73 ± 1.20 B a
N4	4.84 ± 0.11 A c	24.77 ± 1.18 A a	24.74 ± 0.91 Aa	4.79 ± 0.19 A a	0.38 ± 0.01 A c	1.74 ± 0.01 B b	7.28 ± 0.15 A a	30.95 ± 1.69 A a
P4	3.53 ± 0.03 B c	24.14 ± 1.33 A a	23.71 ± 0.23 Ab	1.70 ± 0.30 B a b	0.22 ± 0.02 B b	2.65 ± 0.15 A c	6.43 ± 0.59 A a	3.12 ± 0.10 B b

Data are presented as (mean ± standard error and significance of difference labels) (*n* = 3). Different capital letters indicated significant differences between NF (natural forests) and PF (planted forests), and different lowercase letters indicated significant differences between different growth stages (*p* < 0.05).

**Table 2:** Microbial entropy and stoichiometric imbalance in rhizosphere soil.

Forest Stands	Microbial Entropy			Stoichiometric Imbalance		
	qMBC	qMBN	qMBP	(C/N)_imb_	(C/P)_imb_	(N/P) _imb_
N1	11.74 ± 0.46 A c	7.54 ± 0.59 A a	7.89 ± 0.22 A d	0.64 ± 0.04 A a	0.67 ± 0.02 A b	1.05 ± 0.09 A c
P1	12.01 ± 0.11 A a	5.27 ± 0.45 B a b	6.33 ± 0.42 B b	0.44 ± 0.04 B a	0.53 ± 0.04 B b	1.21 ± 0.10 A b
N2	18.70 ± 1.76 A a	6.62 ± 0.77 A a	21.00 ± 0.53 A c	0.36 ± 0.05 B b	1.13 ± 0.09 A b	3.22 ± 0.43 A b c
P2	6.86 ± 1.69 B b	3.86 ± 0.94 B b c	10.61 ± 2.06 B a	0.59 ± 0.18 A a	1.67 ± 0.64 A a	2.81 ± 0.47 A a
N3	14.70 ± 1.66 A b c	5.16 ± 1.06 A b	25.53 ± 3.53 A b	0.35 ± 0.07 B b	1.77 ± 0.41 A a	5.22 ± 1.72 A a
P3	4.75 ± 1.11 B c	3.31 ± 0.49 B c	8.96 ± 1.25 B a b	0.73 ± 0.16 A a	1.99 ± 0.58 A a	2.74 ± 0.38 B a
N4	15.87 ± 0.99 A a b	7.20 ± 0.59 A a	34.00 ± 1.17 A a	0.46 ± 0.03 B b	2.15 ± 0.08 A a	4.74 ± 0.41 A a b
P4	7.19 ± 0.41 B b c	5.52 ± 0.97 A a	1.55 ± 0.04 B c	0.77 ± 0.12 A a	0.21 ± 0.01 B b	0.29 ± 0.06 B c

Data are presented as (mean ± standard error and significance of difference labels) (*n* = 3). Different capital letters indicate significant differences between NF and PF, and different lowercase letters indicate significant differences between different growth stages (*p* < 0.05).

**Table 3:** Information on experimental plots and diameter-at-breast-height classes.

Stand Types	Stand Plots	Altitude (m)	Aspect of Slope	Slope (°)	Growth Stage (DBH Grade)	Total Number of Trees
Natural forests (NF)	NF1	397.2	E	45~50	N1 (2.5 cm ≤ DBH < 10.5 cm)	28
					N2 (10.5 cm ≤ DBH < 18.5 cm)	27
					N3 (18.5 cm ≤ DBH < 26.5 cm)	24
					N4 (26.5 cm ≤ DBH < 34.5 cm)	16
	NF2	375.4	E	55~60	N1 (2.5 cm ≤ DBH < 10.5 cm)	27
					N2 (10.5 cm ≤ DBH < 18.5 cm)	26
					N3 (18.5 cm ≤ DBH < 26.5 cm)	22
					N4 (26.5 cm ≤ DBH < 34.5 cm)	18
	NF3	366.3	SE	45~50	N1 (2.5 cm ≤ DBH < 10.5 cm)	30
					N2 (10.5 cm ≤ DBH < 18.5 cm)	25
					N3 (18.5 cm ≤ DBH < 26.5 cm)	23
					N4 (26.5 cm ≤ DBH < 34.5 cm)	15
Planted forests (PF)	PF1	144.1	E	10~25	P1 (2.5 cm ≤ DBH < 10.5 cm)	31
					P2 (10.5 cm ≤ DBH < 18.5 cm)	29
					P3 (18.5 cm ≤ DBH < 26.5 cm)	27
					P4 (26.5 cm ≤ DBH < 34.5 cm)	22
	PF2	137.5	SW	10~20	P1 (2.5 cm ≤ DBH < 10.5 cm)	30
					P2 (10.5 cm ≤ DBH < 18.5 cm)	27
					P3 (18.5 cm ≤ DBH < 26.5 cm)	28
					P4 (26.5 cm ≤ DBH < 34.5 cm)	24
	PF3	140.7	NE	10~20	P1 (2.5 cm ≤ DBH < 10.5 cm)	31
					P2 (10.5 cm ≤ DBH < 18.5 cm)	26
					P3 (18.5 cm ≤ DBH < 26.5 cm)	28
					P4 (26.5 cm ≤ DBH < 34.5 cm)	20

Note: The stand density of natural forests is 2400 trees per hectare (including naturally regenerated seedlings), while the management density of planted forests is 1665 trees per hectare.

## Data Availability

The data supporting the results of this study and its analytical procedures are available from the corresponding author upon reasonable request.

## Supplementary Materials

The following supporting information can be downloaded at: https://www.mdpi.com/article/10.3390/plants15213275/s1, Figure S1: Schematic diagram of the experimental design and sample collection; Table S1: Two-way ANOVA analysis of soil physicochemical properties; Table S2. Two-way ANOVA analysis of soil microbial biomass; Table S3. Two-way ANOVA analysis of soil enzyme activity; Table S4. Two-way ANOVA analysis of soil microbial entropy and stoichiometric imbalance; Table S5. Two-way PERMANOVA analysis for rhizosphere fungal and bacterial community composition; Table S6. PERMDISP multivariate dispersion test.
