# Supplementary material for: Ecological Stoichiometry and Microbial Community Dynamics in the Rhizosphere of Parashorea chinensis: Implications for Ex Situ Conservation of Endangered Trees
Source: Plants (Basel). 2026 Oct 23;15(21):3275. doi: 10.3390/plants15213275 (PMC13765806; doi:10.3390/plants15213275)
Supplement: Supplementary file 1 [file plants-15-03275-s001.zip › plants-4627692-supplementary.pdf]

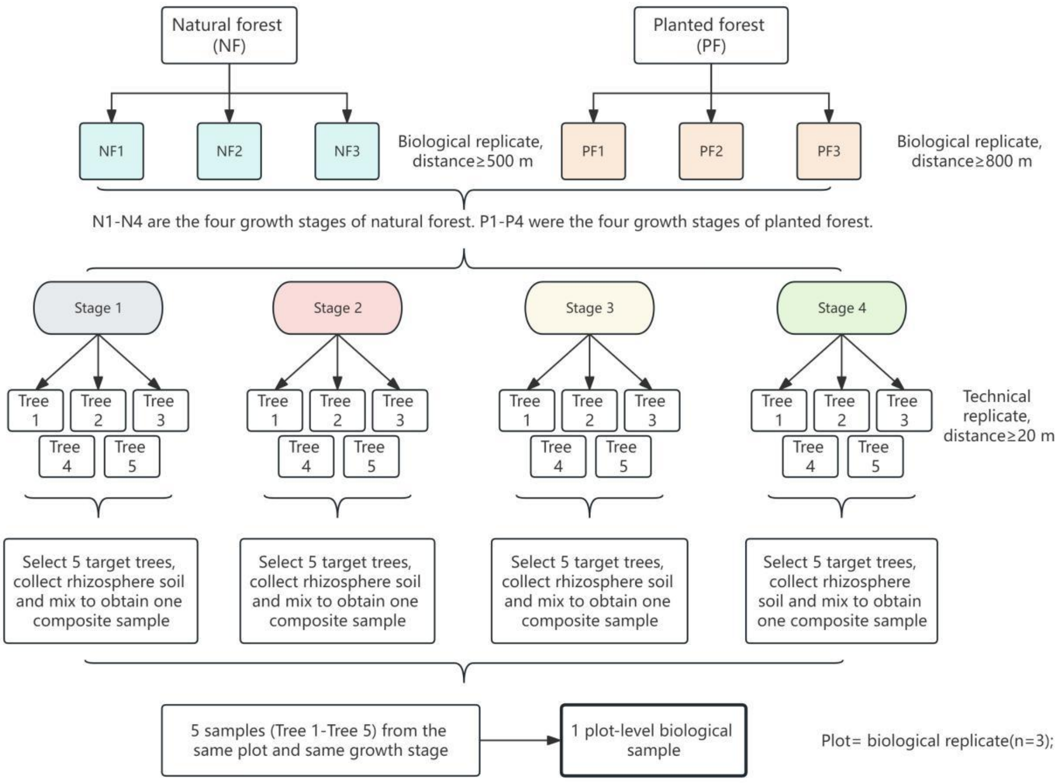

Figure S1. Schematic diagram of the experimental design and sample collection.

Two-way ANOVA results (Table S1) showed that growth stage, forest type, and their interaction exerted highly significant effects on most soil physicochemical properties ($P < 0.001$). Growth stage significantly regulated pH, SWC, SOC, TN, TP, AP, NO_3^--N and NH_4^+-N. Forest type exhibited highly significant effects on all indicators except NO_3^--N ($P = 0.037$**). The interaction between growth stage and forest type had highly significant influences on all measured soil variables. Based on F-values and effect sizes ω^2 and ω^2P, growth stage contributed the greatest variation in NH_4^+-N, while the interactive effect dominated the variation in pH and TN. By contrast, forest type generally explained a low proportion of the total variation for all soil indicators. These findings demonstrated that the rhizosphere soil physicochemical characteristics of *Parashorea chinensis* were jointly modulated by forest type and plant developmental stage, with strong interactive effects between the two factors.

Table S1. Two-way ANOVA analysis of soil physicochemical properties.

**Indexes**	**Sources of Variation**	**Results of Two-Way Anova Test**				**Effect Size**	
		**Quadratic sum**	**Mean Square**	***F*-value**	***P*-value**	**ω^2 Omega-Squared**	**ω^2P Partial Omega-Squared**
pH	Growth Stage	60.033	8.576	1350.621	<0.0001***	**0.0020**	**0.3837**
	Forest Type	3.68	3.68	579.506	<0.0001***	2.9096×10^{-18}	9.0733×10^{-16}
	Interaction	550.76	78.68	12391.041	<0.0001***	0.0018	0.3535
SWC	Growth Stage	464.051	66.293	68.443	<0.0001***	**0.0040**	**0.2306**
	Forest Type	313.645	313.645	323.815	<0.0001***	1.2255×10^{-17}	9.1290×10^{-16}
	Interaction	8431.642	1204.52	1243.578	<0.0001***	0.00364	0.21318
SOC	Growth Stage	8930.179	1275.74	2577.019	<0.0001***	**2.2204×10^{-4}**	**0.2116**
	Forest Type	1632.307	1632.307	3297.292	<0.0001***	2.3627×10^{-19}	2.8558×10^{-16}
	Interaction	19726.286	2818.041	5692.496	<0.0001***	1.9561×10^{-4}	0.1912
TN	Growth Stage	49.465	7.066	79.982	<0.0001***	0.0146	0.3840
	Forest Type	5.716	5.716	64.702	<0.0001***	1.5083×10^{-17}	6.4618×10^{-16}
	Interaction	198.88	28.411	321.578	<0.0001***	**0.0149**	**0.3902**
TP	Growth Stage	0.473	0.068	274.683	<0.0001***	**8.1683×10^{-5}**	**0.0159**
	Forest Type	0.108	0.108	438.723	<0.0001***	1.1535×10^{-18}	2.2825×10^{-16}
	Interaction	6.343	0.906	3684.834	<0.0001***	5.2593×10^{-5}	0.0103
AP	Growth Stage	117.307	16.758	115.801	<0.0001***	**3.8346×10^{-4}**	**0.0132**
	Forest Type	46.683	46.683	322.588	<0.0001***	-5.6096×10^{-18}	-1.9541×10^{-16}
	Interaction	315.096	45.014	311.05	<0.0001***	3.8279×10^{-4}	0.0132
NO_3^--N	Growth Stage	264.836	37.834	268.113	<0.0001***	**4.2435×10^{-4}**	**0.0403**
	Forest Type	0.729	0.729	5.164	=0.0370**	-2.0814×10^{-18}	-2.0571×10^{-16}
	Interaction	1274.34	182.049	1290.106	<0.0001***	2.7492×10^{-4}	0.0265
NH_4^+-N	Growth Stage	3330.886	475.841	120.828	<0.0001***	0.0177	0.7784
	Forest Type	1840.8	1840.8	467.424	<0.0001***	1.0295×10^{-17}	2.0431×10^{-15}
	Interaction	6883.03	983.29	249.681	<0.0001***	**0.0178**	**0.7789**

Note: In two-way ANOVA, the effect sizes ω^2 (omega-squared) and ω^2P (partial omega-squared) quantify the proportion of variance in the dependent variable explained by independent variables to evaluate their influences. ω^2 estimates the explained variance of the two factors and their interaction, yielding robust effect-size estimates. ω^2P precisely assesses the effect of a specific factor under complex experimental designs after accounting for other factors and their interaction. Larger F-values and ω^2 indicate greater contributions of the factor to the variation in observations. Interaction: Growth stage × Forest type interaction. *** and ** denote significance at $P < 0.001$ and $P < 0.01$, respectively. Bold values indicate that the corresponding variable contributed the highest proportion to the variation in the target index (n=3).

Two-way ANOVA results (Table S2) showed that growth stage, forest type, and their interaction exerted highly significant effects on soil microbial biomass carbon (MBC), nitrogen (MBN), and phosphorus (MBP) ($P < 0.001$). In terms of F-values and partial ω^2 effect sizes, growth stage exhibited strong explanatory power for MBC, MBN,

and MBP. The interaction between growth stage and forest type was also highly significant, contributing substantially to the variation in the three microbial biomass indices. Although the main effect of forest type was significant, its effect size was extremely small, indicating a weak standalone explanation for the variation in microbial biomass. Collectively, the rhizosphere soil microbial biomass of *Parashorea chinensis* was primarily regulated by plant growth stage. Forest type also exerted an independent significant effect, and strong interactive effects existed between growth stage and forest type, which together determined the variation patterns of microbial biomass.

Table S2. Two-way ANOVA analysis of soil microbial biomass.

Indexes	Results of Two-Way ANOVA Test					Effect Size	
	**Sources of Variation**	**Quadratic Sum**	**Mean Square**	***F*-value**	***P*-value**	**ω² Omega-Squared**	**ω²P Partial Omega-Squared**
MBC	Growth Stage	243469.539	34781.363	398.929	<0.0001***	**0.0039**	**0.4678**
	Forest Type	20512.56	20512.56	235.271	<0.0001***	1.3617×10^{-18}	3.1000×10^{-16}
	Interaction	1308205.331	186886.476	2143.516	<0.0001***	0.0035	0.4449
MBN	Growth Stage	2367.044	338.149	173.939	<0.0001***	**0.0084**	**0.5780**
	Forest Type	1010.964	1010.964	520.025	<0.0001***	9.3857×10^{-18}	1.5271×10^{-15}
	Interaction	7032.32	1004.617	516.76	<0.0001***	0.0084	0.5768
MBP	Growth Stage	716.323	102.332	275.581	<0.0001***	**0.0048**	**0.4875**
	Forest Type	177.436	177.436	477.839	<0.0001***	6.6557×10^{-18}	1.3145×10^{-15}
	Interaction	1376.849	196.693	529.696	<0.0001***	0.0045	0.4678

Two-way ANOVA results (Table S3) showed that growth stage exerted highly significant effects on the activities of four soil extracellular enzymes (BGA, SUA, URA, APA) ($P < 0.001$). Forest type had highly significant effects on SUA, URA and APA ($P < 0.001$), whereas no significant effect was detected for BGA ($P = 0.172$). The interaction between growth stage and forest type exhibited highly significant influences on SUA, URA and APA ($P < 0.001$), while the interactive effect was non-significant for BGA. Based on F-values and effect sizes ω^2 and ω^2P, growth stage served as the dominant factor governing the variation in URA activity; the activities of the remaining enzymes were also primarily regulated by growth stage, and the interactive effect further explained a portion of their variation.

Table S3. Two-way ANOVA analysis of soil enzyme activity.

Indexes	Results of Two-Way ANOVA Test					Effect Size	
	**Sources of Variation**	**Quadratic Sum**	**Mean Square**	***F*-value**	***P*-value**	**ω² Omega-Squared**	**ω²P Partial Omega-Squared**
BGA	Growth Stage	22565.594	3223.656	96.948	<0.0001***	0.0133	0.2992
	Forest Type	68.092	68.092	2.048	<0.0001***	-5.0046×10^{-18}	-1.6106×10^{-16}
	Interaction	130485.665	18640.809	560.6	=0.172	**0.0158**	**0.3365**
SUA	Growth Stage	1243818.397	177688.342	2401.032	<0.0001***	2.0940×10^{-4}	0.0946
	Forest Type	459023.427	459023.427	6202.603	<0.0001***	1.3225×10^{-18}	6.5983×10^{-16}
	Interaction	6839444.834	977063.548	13202.674	<0.0001***	$\mathbf{2.9134\times10^{-4}}$	**0.1269**
URA	Growth Stage	1965.643	280.806	210.083	<0.0001***	0.0136	0.9323
	Forest Type	97.286	97.286	72.783	<0.0001***	2.3119×10^{-18}	2.3463×10^{-15}
	Interaction	12671.459	1810.208	1354.291	<0.0001***	**0.0137**	**0.9328**
APA	Growth Stage	1555.05	222.15	254.45	<0.0001***	0.0017	0.0748
	Forest Type	173.888	173.888	199.171	<0.0001***	9.1543×10^{-18}	4.2474×10^{-16}
	Interaction	19611.265	2801.609	3208.952	<0.0001***	**0.0024**	**0.1018**

Two-way ANOVA revealed that both forest type and growth stage significantly affected microbial entropy (qMBC, qMBN, qMBP) and stoichiometric imbalance (C/N)imb, (C/P)imb, (N/P)imb ($P < 0.001$). A significant interactive effect between forest type and growth stage was also detected for all these variables ($P < 0.001$). Partial omega-squared values showed that growth stage explained the largest proportion of variance in ((N/P)imb, while forest type exerted strong influences on microbial entropy metrics.

Table S4. Two-way ANOVA analysis of soil microbial entropy and stoichiometric imbalance.

**Indexes**	**Results of Two-Way ANOVA Test**					**Effect Size**	
	**Sources of Variation**	**Quadratic Sum**	**Mean Square**	***F*-value**	***P*-value**	**ω² Omega-Squared**	**ω²P Partial Omega-Squared**
qMBC	Growth Stage	707.096	101.014	128.887	<0.0001***	**0.0050**	**0.2065**
	Forest Type	240.825	240.825	307.276	<0.0001***	1.0364×10^{-17}	5.3557×10^{-16}
	Interaction	3331.716	475.959	607.292	<0.0001***	0.0047	0.1963
qMBN	Growth Stage	63.530	9.076	22.953	<0.0001***	**0.0544**	**0.4913**
	Forest Type	18.716	18.716	47.332	<0.0001***	3.2775×10^{-17}	5.8219×10^{-16}
	Interaction	768.166	109.738	277.529	<0.0001***	0.0538	0.4888
qMBP	Growth Stage	3231.917	461.702	275.526	<0.0001***	**0.0069**	**0.6853**
	Forest Type	1002.352	1002.352	598.165	<0.0001***	4.0505×10^{-18}	1.2703×10^{-15}
	Interaction	6135.431	876.49	523.056	<0.0001***	0.0064	0.6668
(C/N)imb	Growth Stage	0.608	0.087	11.453	<0.0001***	**0.0093**	**0.0529**
	Forest Type	0.246	0.246	32.473	<0.0001***	6.1720×10^{-17}	3.6946×10^{-16}
	Interaction	7.328	1.047	138.046	<0.0001***	0.0085	0.0485
(C/P)imb	Growth Stage	18.260	2.609	27.580	<0.0001***	**0.0126**	**0.1061**
	Forest Type	0.042	0.042	0.442	0.516	1.7470×10^{-17}	1.6414×10^{-16}
	Interaction	48.151	6.879	72.725	<0.0001***	0.0126	0.1041
(N/P) imb	Growth Sage	91.495	13.071	30.766	<0.0001***	**0.0838**	**0.8829**
	Forest Type	10.395	10.395	24.469	<0.0001***	2.4858×10^{-17}	2.2353×10^{-15}
	Interaction	209.469	29.924	70.435	<0.0001***	0.0782	0.8755

For the fungal community, only the interaction between forest type and growth stage was significant ($R^2 = 0.364$, $P = 0.003$), while the main effects of forest type ($P = 0.100$) and growth stage ($P = 0.180$) were non-significant. This indicates that the variation in fungal community composition was mainly driven by the interactive effect of the two factors. For the bacterial community, both main effects of forest type ($R^2 = 0.326$, $P < 0.001$) and growth stage ($R^2 = 0.373$, $P < 0.001$) were highly significant, whereas their interaction was non-significant ($P = 0.999$).

PERMDISP tests were significant for both fungi ($P = 0.001$) and bacteria ($P < 0.001$), revealing significant differences in within-group dispersion. This suggests that the significant PERMANOVA results may partly arise from differences in within-group dispersion rather than solely from divergence in community composition.

Table S5. Two-way PERMANOVA analysis for rhizosphere fungal and bacterial community composition.

**Source**	**df**	**Fungi**				**Bacteria**			
		Sum Sqs	R^2	*F*	*P*	SumOfSqs	R^2	*F*	*P*
Forest Type (T)	1	0.1608	0.096	4.41	0.100	0.2597	0.326	17.75	**<0.001**
Growth Stage (S)	3	0.3187	0.191	2.91	0.180	0.2971	0.373	6.77	**<0.001**
T×S	3	0.6095	0.364	5.57	**0.003**	0.0060	0.007	0.14	0.999
Residual	16	0.5836	0.349	—	—	0.2341	0.294	—	—
Total	23	1.6725	1.000	—	—	0.7969	1.000	—	—

Note: F-values and P-values are not applicable for Residual and Total, denoted by "—". SumOfSqs = Sum of Squares. It represents the total variation in community composition explained by the corresponding term.

Table S6. PERMDISP multivariate dispersion test.

**Community**	***F***	***P***
Fungi	3.73	0.001
Bacteria	4.97	<0.001

Note: R^2 is the proportion of community variation explained by each factor; the relative abundance of fungi and bacteria was calculated by Bray–Curtis distance.
